# Association Between Metabolic and Hormonal Derangements and Professional Exposure to Urban Pollution in a High Intensity Traffic Area

**DOI:** 10.3389/fendo.2020.00509

**Published:** 2020-08-11

**Authors:** Alessio Molfino, Maria Ida Amabile, Maurizio Muscaritoli, Annunziata Germano, Rossella Alfano, Cesarina Ramaccini, Alessandra Spagnoli, Liberato Cavaliere, Gianluca Marseglia, Antonio Nardone, Giuseppina Muto, Umberto Carbone, Maria Triassi, Silvana Fiorito

**Affiliations:** ^1^Department of Translational and Precision Medicine, Sapienza University of Rome, Rome, Italy; ^2^Department of Surgical Sciences, Sapienza University of Rome, Rome, Italy; ^3^Department of Public Health, University Federico II, Naples, Italy; ^4^Department of Public Health and Infectious Diseases, Sapienza University of Rome, Rome, Italy; ^5^Institute of Translational Pharmacology, CNR, Rome, Italy

**Keywords:** air pollution, particulate matter, metabolic syndrome, insulin resistance, adiponectin, leptin

## Abstract

**Rationale:** Studies suggest a relation between exposure to air particulate matter (PM)_2.5_ pollution and greater cardiovascular morbidity, as well as increased risk for obesity and diabetes. We aimed to identify association(s) between nutritional and metabolic status and exposure to environmental pollution in a cohort of policemen exposed to high levels of air pollution.

**Methods:** We considered adult municipal policemen, working in an urban area at high-traffic density with documented high levels of air PM_2.5_ (exposed group) compared to non-exposed policemen. Clinical characteristics, including the presence/absence of metabolic syndrome, were recorded, and serum biomarkers, including adiponectin, leptin, and ghrelin, were assessed.

**Results:** One hundred ninety-nine participants were enrolled, 100 in the exposed group and 99 in the non-exposed group. Metabolic syndrome was documented in 32% of exposed group and in 52.5% of non-exposed group (*P* = 0.008). In the exposed group, we found a positive correlation between body mass index and serum leptin as well as in the non-exposed group (*P* < 0.0001). Within the exposed group, subjects with metabolic syndrome showed lower serum adiponectin (*P* < 0.0001) and higher leptin (*P* = 0.002) levels with respect to those without metabolic syndrome, whereas in the non-exposed group, subjects with metabolic syndrome showed only higher leptin levels when compared to those without metabolic syndrome (*P* = 0.01). Among the participants with metabolic syndrome, we found lower adiponectin levels in those of the exposed group with respect to the non-exposed ones (*P* = 0.007). When comparing the exposed and non-exposed groups, after stratifying participants for Homeostatic Model Assessment for Insulin Resistance >2.5, we found lower adiponectin levels in those of the exposed group with respect to the non-exposed ones (*P* = 0.038).

**Conclusions:** Exposure to air PM pollution was associated with lower levels of adiponectin in adult males with metabolic syndrome.

## Introduction

In industrialized countries, air pollution determines risks for human health (1). Particulate and gaseous emissions from motor vehicles contribute to increase the air pollution (2). Diesel exhaust particles (DEPs), a category of particulate matter (PM) derived from diesel fossil fuels and combustible engines, are among the most abundant components of airborne PM with an aerodynamic diameter <2.5 μm (PM_2.5_) (1) and are considered major contributors to traffic-related PM in urban areas (2, 3). The most part of DEP is composed by particles of nano-sized dimension (4), which are small aggregates of carbonaceous particles (<100 nm), representing a severe problem for human health considering that they remain in the atmosphere for long periods and go beyond to the indoor air environment, and they can be breathed determining a more toxic effect deeply into the lungs with respect to the coarse particles (5).

Particulate pollution is a major public health concern. Several epidemiological studies demonstrated that exposure to DEP is related to various cardiopulmonary, vascular, and oncologic diseases (6–9). Moreover, carbonaceous nanoparticles derived from diesel engine exhaust are classified as human carcinogens (10, 11). Recent data from large cohorts of subjects have shown strong associations between exposure to air PM_2.5_ pollution and increased cardiovascular morbidity and mortality (12, 13). Long-term air pollution exposure to fine PM_2.5_ above US Environmental Protection Agency–defined standards has been reported to be associated with higher risk of neurodegenerative diseases, including Alzheimer disease (14).

Moreover, it has been reported that exposure to diesel exhaust PM may increase the risk for obesity and diabetes (15–17). Recently, it has been demonstrated that an early exposure to high levels of PM_2.5_ during life represents a risk factor for development of adiposity and insulin resistance in the subsequent years, likely mediated at least in part by reactive oxygen species generation (16). Exposure for a long period of time to ultrafine particles in areas near highways has been associated with stroke, ischemic heart disease, hypertension, and type 2 diabetes (17, 18). Robust data highlighted the association between long-term exposure to air pollution and type 2 diabetes and neurodegenerative disorders in adults, such as dementia and a general decline in cognition (19). Another recent study identified the alteration of several metabolic pathways that mediate the development of asthma and cardiovascular diseases associated with ambient air pollutants (20). Moreover, a study conducted in non-obese children exposed to high concentrations of PM_2.5_, vs. low pollution controls, showed high circulating leptin and endothelin 1 levels, vitamin D deficiency, and food reward hormone dysregulation (21).

At present, few data are available on the association between hormone dysregulation, metabolic derangement, and chronic exposure to environmental contaminants.

By the present study, we aimed at identifying association(s) between nutritional, metabolic, and hormonal derangements and exposure to environmental pollution in an Italian population of traffic policemen professionally exposed to high levels of air PM_2.5_ compared to employees performing indoor administrative work in the same area.

## Materials and Methods

This is a cross-sectional study performed on adult male municipal policemen of the city of Naples, Italy. The male gender was chosen because of the high percentage of male subjects working in this field in this specific geographical area.

This study was carried out in accordance with the health surveillance program (D.L. n.81/08), approved to be conducted at the Department of Public Health, University of Naples “Federico II,” Italy. All the subjects gave written informed consent in accordance with the Declaration of Helsinki and authorized the use of the clinical data for research purposes. The privacy rights of human subjects were always observed. Exclusion criteria included the presence of highly catabolic diseases, such as cancer, chronic infections, and the absence of informed consent.

### Participant Demographic and Clinical Characteristics

We enrolled adult males professionally exposed to air pollution (or airborne nanoparticles) in an urban area at high traffic density (exposed group) and adult males non-exposed (at least 1 year indoor working) (non-exposed group) matched by body mass index (BMI).

Demographic characteristics, including age, weight, height, BMI, and comorbidities, such as hypertension, diabetes, cardiovascular disorders, and dyslipidemia, were recorded in all participants. The diagnostic criteria we used for metabolic syndrome were based on the National Cholesterol Education Program Adult Treatment Panel III clinical criteria for defining the metabolic syndrome.

### Biomarkers

Blood samples were collected in all the participants on fasting condition and then centrifuged, and the serum was stored at −80°C at the Laboratory of the Department of Public Health, University of Naples “Federico II.” The samples were shipped to the Central Laboratory for analysis at the Department of Translational and Precision Medicine (formerly Department of Clinical Medicine), Sapienza University of Rome. The glycemic–insulinemic profile, including the Homeostatic Model Assessment for Insulin Resistance (HOMA-IR) index and the complete lipid profile (total cholesterol, high-density lipoprotein, low-density lipoprotein, triglycerides), has been assessed by enzymatic and/or colorimetric methods.

The serum levels of leptin and adiponectin, as adipokines, and ghrelin, as gastrointestinal peptide, have been assessed by enzyme-linked immunosorbent assays.

We additionally utilized leptin/BMI and adiponectin/BMI ratios in our analyses accounting for the well-known associations between leptin and adiponectin with adiposity, as previously shown (22, 23).

### Statistical Analyses

Participants' characteristics were described using mean ± SD for continuous normally distributed variables, median and interquartile range for non–normally distributed variables, as appropriate, and categorical variables were presented as number of cases (percentages). Skewed variables were transformed to the natural logarithm (LN). A Shapiro–Wilk test was used to determine normality. Relations among variables were assessed through χ^2^ tests, *t*-tests, analysis of variance, or Wilcoxon rank-sum tests, as appropriate. Spearman correlation index was used for non-parametric correlations.

A standard two-tailed *P* < 0.05 was considered statistically significant. All statistical analysis was performed in SPSS®.

## Results

### Characteristics of the Participants

A total of 200 participants were consecutively enrolled in the study during the second half of the year 2016: 100 adult males, who worked as traffic policemen exposed to airborne traffic–derived pollution in an urban area (Naples, Italy) at high traffic density (exposed group) (mean duration of the exposure was 20.2 years, min. 16, max. 29 years), and 100 adult males with administrative duties performed in the office in the same city (non-exposed group). One participant of the non-exposed group was excluded because he did not perform blood sampling and did not respond to the administered questionnaires. Therefore, 99 participants in the non-exposed group were studied.

According to the Air Quality Monitoring Report 2016 by the Regional Agency for Environmental Protection in Campania, Italy, during the 3 months before the enrollment, the traffic policemen were exposed to daily mean PM_2.5_ values between 13.97 and 23.37 μg/m^3^. The same agency recommends maximum values of PM_2.5_ of 25 μg/m^3^ during the entire year (http://www.arpacampania.it/home). No differences were observed between exposed and non-exposed groups in terms of cigarette smoking. In particular, the exposed group included 30 smokers (30%), and 21 of them (70%) smoking more than 10 cigarettes/day. Non-exposed group included 31 smokers (31.3%), and 27 of them (87%) smoking more than 10 cigarettes/day.

### Nutritional and Metabolic Profile of the Participants

No differences were observed between the two groups in terms of body weight, BMI, and lipid profile, whereas plasma glucose levels and HOMA-IR were higher in the non-exposed group compared to the exposed one (*P* = 0.009, *P* = 0.03, respectively) (Table 1). In addition, BMI was ≥30 kg/m^2^ in 22% of the exposed group and in 23.2% of the non-exposed group (Table 1). The presence of comorbidities was as follows: diabetes was present in 7% in the exposed group and in 10.5% of the non-exposed group; hypertension was more frequent in the non-exposed group (*P* = 0.025); dyslipidemia was present in 45% of participants in the exposed group and 43.4% of the non-exposed ones (Table 1). Metabolic syndrome was documented in 32% of the exposed group and in the 52.5% of the non-exposed group (*P* = 0.008) (Table 1). Between the two groups, no differences were seen in terms of adiponectin, ghrelin, and leptin serum levels (Table 1), also when considering these values as ratio with BMI.

**Table 1:** Participants' characteristics.

	**Exposed group**	**Non-exposed group**	***P***
	**Participants *n* = 100**	**Participants *n* = 99**	
Age, years	58.0 (47.75, 61.0)	61.0 (57.0, 63.0)	<0.0001
Body weight, kg	80.0 (75.0, 90.0)	82.5 (75.0, 90.0)	0.52
BMI, kg/m^2^	26.2 (24.5, 28.7)	27.3 (24.9, 29.4)	0.28
BMI ≥30 kg/m^2^, *n* (%)	22 (22%)	23 (23.2%)	0.84
Cholesterol, mg/dL	181.0 (160.8, 213.0)	185.0 (158.5, 209.5)	0.81
LDL cholesterol, mg/dL^*^	116.3 ± 37.1	114.8 ± 35.8	0.78
Triglycerides, mg/dL	116.5 (72.0, 151.8)	107.0 (85.0, 156.0)	0.93
Fasting glucose levels, mg/dL	96.5 (89.0, 106.3)	104.0 (92.0, 116.5)	0.009
Insulinemia, μU/mL	10.5 (6.45, 17.15)	12.5 (7.56, 18.4)	0.10
HOMA-IR index	2.39 (1.46, 4.08)	3.16 (1.81, 5.05)	0.03
**Comorbidities, Yes/No:**			
Hypertension	30/70	46/53	0.025
Diabetes mellitus	7/93	10/89	0.445
Dyslipidemia	34/66	28/71	0.38
Metabolic syndrome	32/68	52/47	0.008
LN adiponectin, ng/mL^*^	11.02 ± 0.5	11.09 ± 0.55	0.39
LN ghrelin, ng/mL^*^	2.22 ± 0.23	2.26 ± 0.20	0.13
Leptin, ng/mL	1.1 (0.63, 2.00)	1.14 (0.56, 1.99)	0.81

*Median (interquartile range) is shown for non–normally distributed variables*.

* *Mean ± SD*.

*BMI, body mass index; LDL, low-density lipoprotein; HOMA-IR, Homeostatic Model Assessment for Insulin Resistance; LN, natural logarithm*.

### Correlation Between BMI and Serum Adiponectin, Ghrelin, and Leptin in the Two Groups of Participants

**Figure 1:**
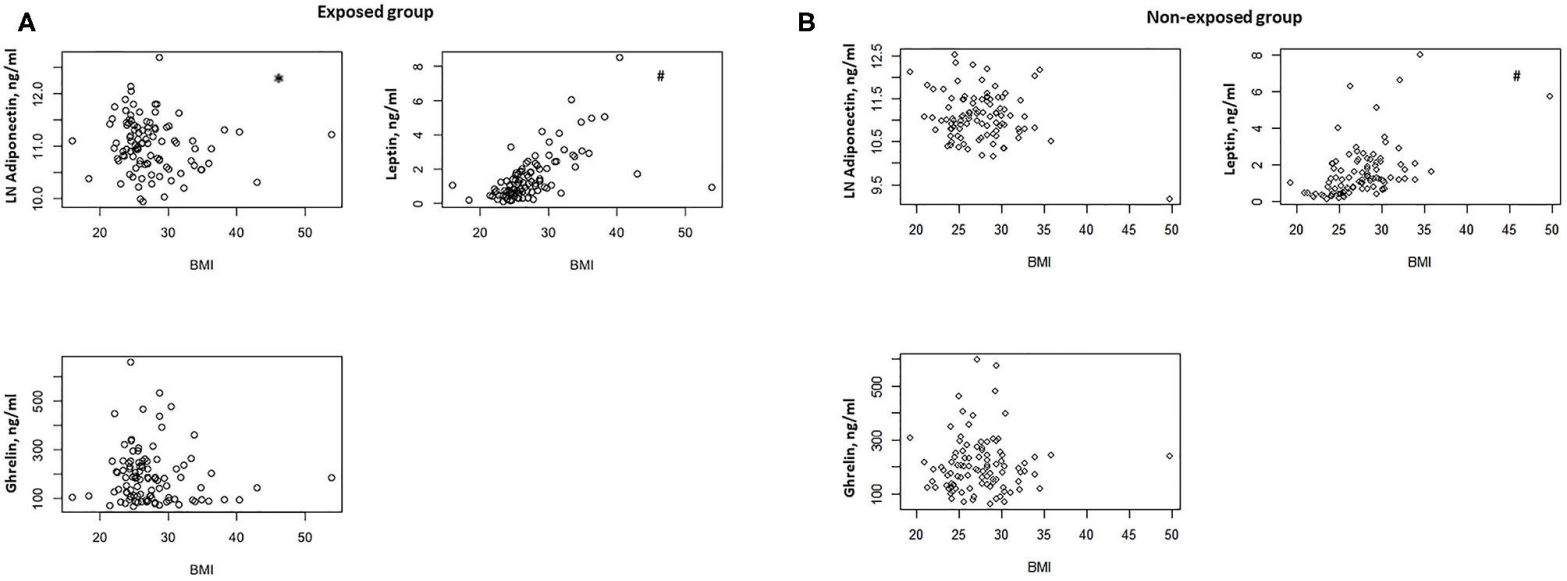
**(A)** Correlation between BMI and serum adiponectin, ghrelin, and leptin levels in the exposed group (*n* = 100). *P = 0.04, ^#^*P* < 0.0001. **(B)** Correlation between BMI and serum adiponectin, ghrelin, and leptin levels in the non-exposed group (*n* = 99). ^#^*P* < 0.0001.

In the exposed group, we found a negative correlation between BMI and serum adiponectin levels (ρ = −0.0205, *P* = 0.04) (Figure 1A) and a positive correlation between BMI and serum leptin concentrations (ρ = 0.667, *P* < 0.0001) (Figure 1A). No significant correlation was documented between BMI and serum ghrelin levels (Figure 1A).

In the non-exposed group, we found a positive correlation between BMI and serum leptin (ρ = 0.546, *P* < 0.0001) (Figure 1B), but no significant correlations were detected between BMI and serum adiponectin and ghrelin (Figure 1B).

### Association Between Comorbidities, Metabolic Syndrome, and Serum Adiponectin, Ghrelin, and Leptin Levels in the Two Groups of Participants

**Figure 2:**
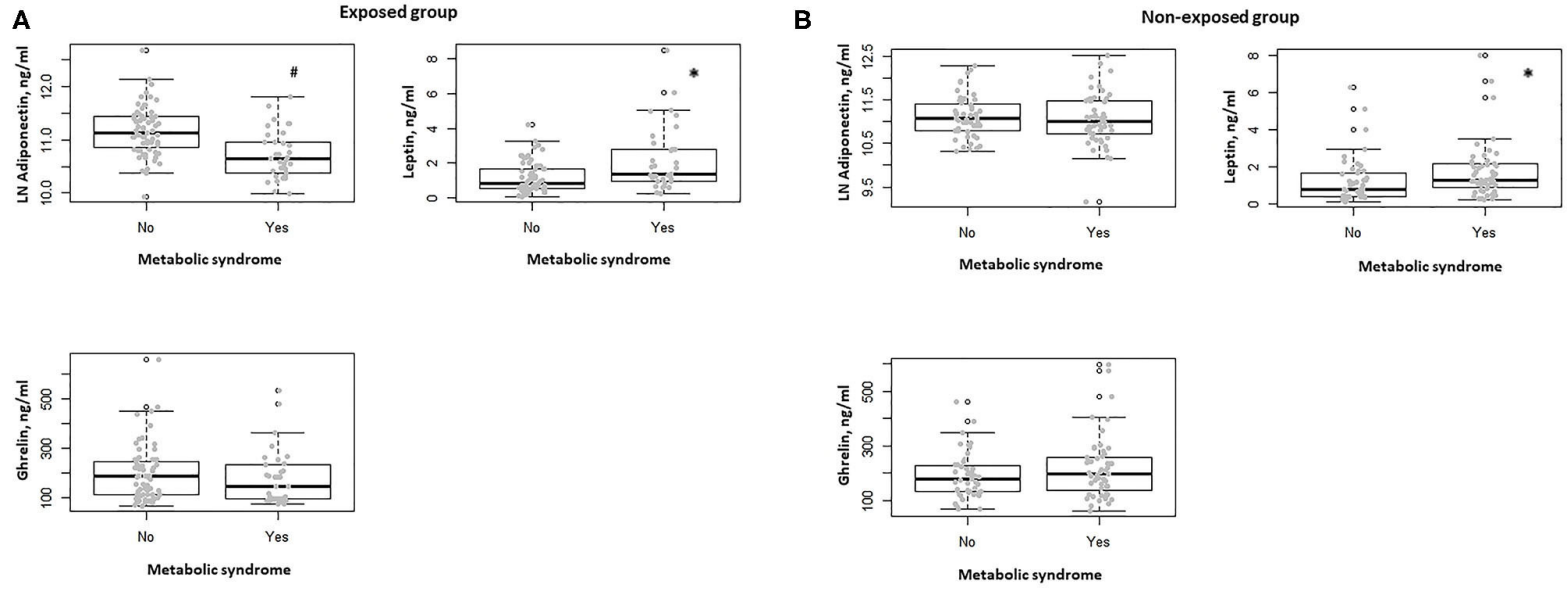
**(A)** Box-dot plot of serum adiponectin, ghrelin, and leptin levels in the exposed group in subjects with (*n* = 32) and without metabolic syndrome (*n* = 68). *P = 0.002, ^#^*P* < 0.0001. **(B)** Box-dot plot of serum adiponectin, ghrelin, and leptin levels in the non-exposed group in subjects with (*n* = 52) and without metabolic syndrome (*n* = 47). *P = 0.01.

In the exposed group, participants with diabetes showed lower serum adiponectin levels with respect to non-diabetic (*P* = 0.001). No association was observed between the other comorbidities, including obesity, hypertension, dyslipidemia, and serum adiponectin, ghrelin, and leptin levels. Subjects in this group with metabolic syndrome showed lower serum adiponectin levels and higher serum leptin levels with respect to those without metabolic syndrome (*P* < 0.0001, *P* = 0.002, respectively) (Figure 2A). This observation was confirmed when correcting per BMI both serum levels of adiponectin (adiponectin/BMI) (*P* < 0.0001) and leptin (leptin/BMI) (*P* = 0.003).

In the non-exposed group, no differences in serum adiponectin, ghrelin, and leptin levels were documented between diabetic and non-diabetic participants and when considering the presence of obesity, hypertension, and dyslipidemia. In the non-exposed subjects with metabolic syndrome, we found no significant difference in terms of serum adiponectin levels, whereas higher serum leptin levels were observed when compared to those without metabolic syndrome (*P* = 0.58, *P* = 0.01, respectively) (Figure 2B). Also, in this group, this behavior was confirmed when correcting per BMI both serum levels of adiponectin (adiponectin/BMI) (*P* < 0.0001) and leptin (leptin/BMI) (*P* = 0.018).

### Metabolic Syndrome and Metabolic Biomarkers Between the Two Groups of Participants

**Figure 3:**
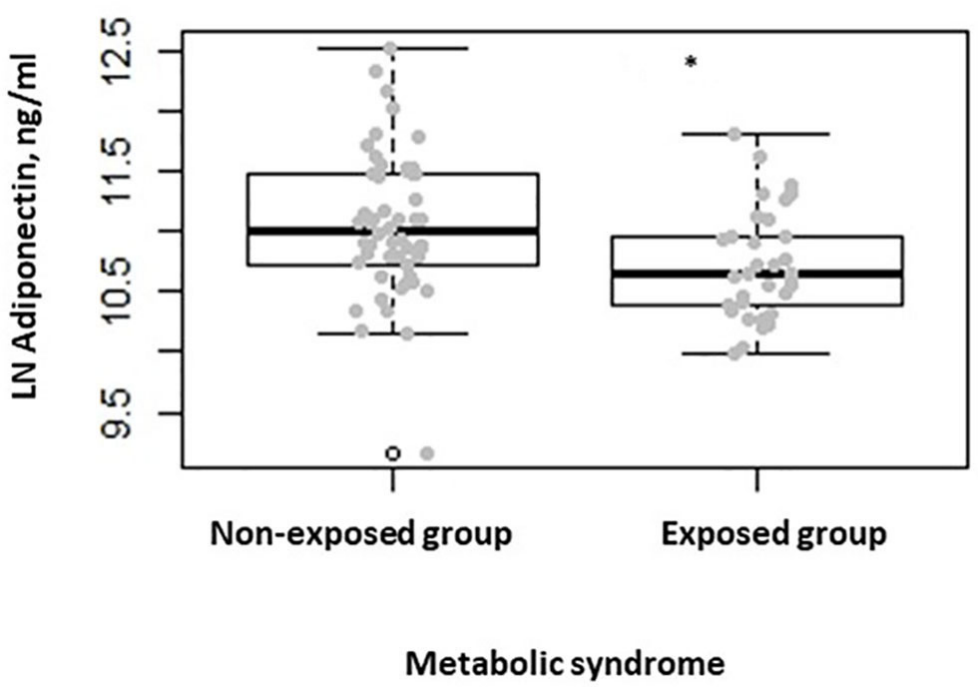
Box-dot plot of serum adiponectin levels in subjects with metabolic syndrome of the non-exposed (*n* = 52) and of the exposed (*n* = 32) group (*P = 0.007).

When comparing the two groups after stratifying participants for the presence of metabolic syndrome, we found lower serum adiponectin levels in the exposed group when compared to the non-exposed one (*P* = 0.007) (Figure 3). No differences were seen in terms of serum leptin, ghrelin, insulin, and fasting plasma glucose levels. Finally, when comparing the two groups, after stratifying participants for HOMA-IR > 2.5, as indicator of insulin resistance (24), we found lower adiponectin serum levels in the exposed group with respect to the non-exposed (*P* = 0.038).

## Discussion

A large number of studies performed worldwide have shown that environmental pollution due to exhaust particles and gas from diesel engine vehicles heavily impacts human health (1–3, 9, 10). In the last 10 years, several scientific reports highlighted the dangerous effects for human health deriving from the exposure to DEP. The contact of micro (PM 10–2.5 μm) and/or nano (PM < 100 nm) sized particles with mucous membranes of the respiratory systems through breathing, the potential achievement of the brain tissue through nasal inhalation, and the direct absorption through the skin have been considered the main entrance doors of the particles into the human body. Several data both *in vitro* and *in vivo* have documented the deleterious effects of DEP. The impact on different cell systems has been assessed, as well as the underlying cellular mechanisms regulating the biological effects between pollution-derived particles (25–30). It has been widely demonstrated that the biological effects of urban traffic pollution are mediated by inflammatory mechanisms.

The results of our study, aimed at investigating the potential effects of environmental pollutants on metabolic and hormonal parameters in people exposed professionally to urban traffic, showed that, when comparing the two groups of participants (exposed vs. non-exposed), no differences were detectable in terms of serum adiponectin, ghrelin, and leptin levels. However, in the two groups we confirmed the presence of a positive correlation between BMI and leptin concentrations, as expected to be influenced by adiposity, while the negative correlation between BMI and adiponectin was present only in the exposed group, where also the presence of diabetes was associated with lower serum adiponectin levels. A similar behavior was shown regarding the association between metabolic syndrome and changes in terms of both adiponectin (lower) and leptin (higher) circulating levels only in the exposed group; in fact, in the non-exposed subjects, only leptin serum levels were higher in subjects with metabolic syndrome. More importantly, when comparing the two groups after stratifying participants for the presence of metabolic syndrome, as well as for HOMA-IR > 2.5 alone, we found significantly lower serum adiponectin levels in the exposed group with respect to the non-exposed. Among the metabolic and hormonal parameters (insulin, ghrelin, and leptin), our data suggest that the prolonged daily exposure to high levels of urban pollution, as documented by the report of the Regional Agency for Environmental Protection, significantly affected only the adiponectin serum levels.

Adiponectin is a 244-amino-acid protein, produced almost exclusively by white adipocyte cells, mostly by visceral depots (31); it possesses a wide range of biological activities, mainly including an insulin-sensitizing and antiatherogenic function, and has been identified as a potent and pleiotropic regulator of inflammation (32–35). Experimental studies have provided strong evidences that major cellular responses to PM exposure include oxidative stress and inflammation (36, 37). The majority of the effects induced by exhaust emissions determine the triggering of inflammatory responses in exposed individuals and the gradual release of cytokines, reactive oxygen species, nitric oxide synthase, and other defense mechanisms, which ultimately expose the DNA to negative oxidative effects (38). Moreover, several recent studies highlighted the fundamental role played by PM exposure in the pathogenesis of the metabolic syndrome and cardiovascular disorders (39). Increased PM_2.5_ exposure has been shown to promote elevations in blood pressure among healthy subjects and in particular among obese individuals who resulted more susceptible to the effects of ambient air pollution (40). High levels of air PM with diameter ≤10 and 2.5 μm have been demonstrated to impair myocardial perfusion and increase myocardial oxygen demand in non-smoking patients with metabolic syndrome (41). Air pollution has been implicated in the pathogenesis of metabolic syndrome associated with other metabolic disorders (42, 43).

A hormonal derangement, likely triggering the onset of the metabolic syndrome during prolonged exposure to PM_10_, has been reported (44).

Our findings on reduced adiponectin serum levels in exposed subjects with metabolic syndrome are supported by a recent study reporting higher levels of cadherin 13 upon exposure to PM_10_ that, resulting in reduced levels of free adiponectin, could affect insulin resistance, as adiponectin is crucial to its reduction. The authors suggested that long-term exposure to PM_10_ may interfere with insulin metabolism, as adiponectin plays a central role in regulating insulin levels (45, 46).

A more recent study showed that exposure to 1-year average PM_2.5_ is associated with an increased risk of metabolic syndrome and its components in adults without cardiovascular disease, thus indicating that PM_2.5_ affects the onset of metabolic syndrome, which may lead to increase the risk of cardiovascular disease (47). A significant association of short- and long-term exposures to PM_2.5_ with hypertension has been observed with a stronger relationship among studies of men from Asia, North America, and areas with higher air pollutant levels (48).

Regarding our study, we should consider that the city of Naples is on the seaside, exposed during almost all hours of the day to the sea breeze or wind. It is likely that these windy atmospheric conditions are able to disperse gaseous and particulate exhaust components decreasing considerably their inhaled portion, thus potentially reducing their dangerous effects. In this light, the metabolic derangements that we observed in a small percentage (32%) of our exposed group might be even worse in a more polluted metropolitan area.

Our study has limitations, including the absence of an objective assessment of physical activity/inactivity, in particular in the non-exposed group. However, we assume that the exposed group is more physically active that the non-exposed one at least during the working time.

We did not assess food intake, which might help in correlating metabolic and hormonal changes with energy and protein imbalance. To account for adiposity, we did not measure waist circumference, as well as body composition, although we corrected the levels of the biomarkers for body size (BMI).

We considered only male subjects, and more importantly, we are not able to ascertain the effect of a single subject's exposure to pollution and to cigarettes smoking, as potentially additive effects, and the change in hormonal and metabolic profile in a longitudinal fashion. Moreover, a higher percentage of hypertensive and metabolic syndrome individuals in the non-exposed cohort might have impacted on the correlations observed. However, considering that the exposed group is the one showing significantly higher metabolic dysregulations in terms of leptin and adiponectin levels, we believe that the data obtained within this group appear reliable, although a cause-effect investigation is needed to clarify these observations.

In conclusion, the significantly lower circulating adiponectin levels documented in the exposed group with metabolic syndrome appear clinically important, suggesting that the exposed group, despite protective factors such as non-sedentary daily activity, is subjected to undergo metabolic changes likely due to the observed reduced levels of the hormone adiponectin (which possesses a protective function against inflammation) potentially induced by environmental pollution. Based on these findings, it is possible to hypothesize that being constantly exposed to traffic-derived pollutants may induce metabolic derangements that could lead, if added to other risk factors, including genetic background and unbalanced dietary habits, to the development of chronic diseases including diabetes.

## Data Availability Statement

The datasets generated for this study are available on request to the corresponding author.

## Ethics Statement

Ethical review and approval was not required for the study on human participants in accordance with the local legislation and institutional requirements. The patients/participants provided their written informed consent to participate in this study. Written informed consent was obtained from the individual(s) for the publication of any potentially identifiable images or data included in this article.

## Author Contributions

AM designed research, analyzed data, and wrote the paper. MA collected the data and wrote the paper. MM and UC reviewed the paper. AG and RA conducted research and collected the data. CR performed laboratory dosage. AS performed statistical analyses. LC, GMa, AN, and GMu collected the data and reviewed the paper. MT conducted the research and reviewed the paper. SF designed research, wrote the paper, and had primary responsibility for final content. All authors contributed to the article and approved the submitted version.

## Conflict of Interest

The authors declare that the research was conducted in the absence of any commercial or financial relationships that could be construed as a potential conflict of interest. The handling Editor declared a shared affiliation, though no other collaboration, with the authors AM, MA, MM, CR, and AS at the time of the review.

## References

[1] Mannucci PM Franchini M. Health effects of ambient air pollution in developing countries. Int J Environ Res Public Health. (2017) 14:E1048. 10.3390/ijerph14091048 28895888 PMC5615585

[2] Crinnion W. Particulate matter is a surprisingly common contributor to disease. Integr Med (Encinitas). (2017) 16:8–12. 30881250 PMC6415634

[3] Patel MM Chillrud SN Correa JC Hazi Y Feinberg M Kc D. Traffic-related particulate matter and acute respiratory symptoms among New York city area adolescents. Environ Health Perspect. (2010) 118:1338–43. 10.1289/ehp.0901499 20452882 PMC2944099

[4] Nemmar A Al-Maskari S Ali BH Al-Amri IS. Cardiovascular and lung inflammatory effects induced by systemically administered diesel exhaust particles in rats. Am J Physiol Lung Cell Mol Physiol. (2007) 292:L664–70. 10.1152/ajplung.00240.2006 17085524

[5] Cassee FR Héroux ME Gerlofs-Nijland ME Kelly FJ. Particulate matter beyond mass: recent health evidence on the role of fractions, chemical constituents and sources of emission. Inhal Toxicol. (2013) 25:802–12. 10.3109/08958378.2013.850127 24304307 PMC3886392

[6] Donaldson K Poland CA. Inhaled nanoparticles and lung cancer - what we can learn from conventional particle toxicology. Swiss Med Wkly. (2012) 142:w13547. 10.4414/smw.2012.13547 22714122

[7] Hamanaka RB Mutlu GM. Particulate matter air pollution: effects on the cardiovascular system. Front Endocrinol. (2018) 9:680. 10.3389/fendo.2018.00680 30505291 PMC6250783

[8] Kelly FJ Fussell JC. Air pollution and airway disease. Clin Exp Allergy. (2011) 41:1059–71. 10.1111/j.1365-2222.2011.03776.x 21623970

[9] Shah AS Langrish JP Nair H McAllister DA Hunter AL Donaldson K. Global association of air pollution and heart failure: a systematic review and meta-analysis. Lancet. (2013) 382:1039–48. 10.1016/S0140-6736(13)60898-3 23849322 PMC3809511

[10] Turner MC Krewski D Pope CA 3rd Chen Y Gapstur SM Thun MJ. Long-term ambient fine particulate matter air pollution and lung cancer in a large cohort of never-smokers. Am J Respir Crit Care Med. (2011) 184:1374–81. 10.1164/rccm.201106-1011OC 21980033

[11] IARC (2012). IARC Monographs on Evaluation of Carcinogenic Risks to Humans. Volume 105: Diesel and Gasoline Engine Exhausts and Some Nitroarenes. Available online at: http://www.iarc.fr/en/media-centre/iarcnews/2012/mono105-info.php (accessed June 30, 2019).

[12] Pope CA 3rd Burnett RT Thurston GD Thun MJ Calle EE Krewski D. Cardiovascular mortality and long-term exposure to particulate air pollution: epidemiological evidence of general pathophysiological pathways of disease. Circulation. (2004) 109:71–7. 10.1161/01.CIR.0000108927.80044.7F 14676145

[13] Miller KA Siscovick DS Sheppard L Shepherd K Sullivan JH Anderson GL. Long-term exposure to air pollution and incidence of cardiovascular events in women. N Engl J Med. (2007) 356:447–58. 10.1056/NEJMoa054409 17267905

[14] Calderón-Garcidueñas L Solt AC Henríquez-Roldán C Torres-Jardón R Nuse B Herritt L. Long-term air pollution exposure is associated with neuroinflammation, an altered innate immune response, disruption of the blood-brain barrier, ultrafine particulate deposition, and accumulation of amyloid beta-42 and alpha-synuclein in children and young adults. Toxicol Pathol. (2008) 36:289–310. 10.1177/0192623307313011 18349428

[15] Di Bonaventura M Nicolucci A Meincke H Le Lay A Fournier J. Obesity in Germany and Italy: prevalence, comorbidities, and associations with patient outcomes. Clinicoecon Outcomes Res. (2018) 10:457–75. 10.2147/CEOR.S157673 30197528 PMC6113914

[16] Xu X Yavar Z Verdin M Ying Z Mihai G Kampfrath T. Effect of early particulate air pollution exposure on obesity in mice: role of p47phox. Arterioscler Thromb Vasc Biol. (2010) 30:2518–27. 10.1161/ATVBAHA.110.215350 20864666 PMC3065931

[17] Li Y Lane KJ Corlin L Patton AP Durant JL Thanikachalam M. Association of long-term near-highway exposure to ultrafine particles with cardiovascular diseases, diabetes and hypertension. Int J Environ Res Public Health. (2017) 14:E461. 10.3390/ijerph14050461 28445425 PMC5451912

[18] Toledo-Corral CM Alderete TL Habre R Berhane K Lurmann FW Weigensberg MJ. Effects of air pollution exposure on glucose metabolism in Los Angeles minority children. Pediatr Obes. (2018) 13:54–62. 10.1111/ijpo.12188 27923100 PMC5722706

[19] Dimakakou E Johnston HJ Streftaris G Cherrie JW. Exposure to environmental and occupational particulate air pollution as a potential contributor to neurodegeneration and diabetes: a systematic review of epidemiological research. Int J Environ Res Public Health. (2018) 15:E1704. 10.3390/ijerph15081704 30096929 PMC6121251

[20] Jeong A Fiorito G Keski-Rahkonen P Imboden M Kiss A Robinot N. Perturbation of metabolic pathways mediates the association of air pollutants with asthma and cardiovascular diseases. Environ Int. (2018) 119:334–45. 10.1016/j.envint.2018.06.025 29990954

[21] Calderón-Garcidueñas L Franco-Lira M D'Angiulli A Rodríguez-Díaz J Blaurock-Busch E Busch Y. Mexico City normal weight children exposed to high concentrations of ambient PM2.5 show high blood leptin and endothelin-1, vitamin D deficiency, and food reward hormone dysregulation versus low pollution controls. Relevance for obesity and Alzheimer disease. Environ Res. (2015) 140:579–92. 10.1016/j.envres.2015.05.012 26037109

[22] Lee JH Reed DR Price RA. Leptin resistance is associated with extreme obesity and aggregates in families. Int J Obes Relat Metab Disord. (2001) 25:1471–3. 10.1038/sj.ijo.0801736 11673768

[23] Molfino A Kaysen GA Chertow GM Doyle J Delgado C Dwyer T. Validating appetite assessment tools among patients receiving hemodialysis. J Ren Nutr. (2016) 26:103–10. 10.1053/j.jrn.2015.09.002 26522141 PMC4796001

[24] Molfino A Rossi Fanelli F Laviano A. Sympathetic nervous system activity may link hyperphagia and fat deposition in human obesity. Am J Physiol Endocrinol Metab. (2007) 293:E1129. 10.1152/ajpendo.00500.2007 17911351

[25] Su DS Serafino A Müller JO Jentoft RE Schlögl R Fiorito S. Cytotoxicity and inflammatory potential of soot particles of low-emission diesel engines. Environ Sci Technol. (2008) 42:1761–5. 10.1021/es0716554 18441832

[26] Lawal AO. Diesel exhaust particles and the induction of macrophage activation and dysfunction. Inflammation. (2018) 41:356–63. 10.1007/s10753-017-0682-6 29047037

[27] Tseng CY Wang JS Chao MW. Causation by diesel exhaust particles of endothelial dysfunctions in cytotoxicity, pro-inflammation, permeability, and apoptosis induced by ROS generation. Cardiovasc Toxicol. (2017) 17:384–92. 10.1007/s12012-016-9364-0 26965709

[28] Fiorito S Mastrofrancesco A Cardinali G Rosato E Salsano F Su DS Effects of carbonaceous nanoparticles from low-emission and older diesel engines on human skin cells. Carbon. (2011) 49:5038–48. 10.1016/j.carbon.2011.07.022

[29] Mastrofrancesco A Alfè M Rosato E Gargiulo V Beatrice C Di Blasio G. Proinflammatory effects of diesel exhaust nanoparticles on scleroderma skin cells. J Immunol Res. (2014) 2014:138751 10.1155/2014/138751 24982919 PMC4058589

[30] Steiner S Bisiq C Petri-Fink A Rothen-Rutishauer B. Diesel exhaust: current knowledge of adverse effects and underlying cellular mechanisms. Arch Toxicol. (2016) 90:1541–53. 10.1007/s00204-016-1736-5 27165416 PMC4894930

[31] Arita Y Kihara S Ouchi N Takahashi M Maeda K Miyagawa J. Paradoxical decrease of an adipose-specific protein, adiponectin, in obesity. Biochem Biophys Res Commun. (1999) 257:79–83. 10.1006/bbrc.1999.0255 10092513

[32] Dietz JJ Iglesias P. The role of the novel adipocyte derived hormone adiponectin in human disease. Eur J Endocrinol. (2003) 148:293–300. 10.1530/eje.0.1480293 12611609

[33] Ohashi K Ouchi N Matsuzawa Y. Anti-inflammatory and anti-atherogenic properties of adiponectin. Biochimie. (2012) 94:2137–42. 10.1016/j.biochi.2012.06.008 22713764

[34] Yamauchi T Kamon J Waki H Terauchi Y Kubota N Hara K. The fat-derived hormone adiponectin reverses insulin resistance associated with both lipoatrophy and obesity. Nat Med. (2001) 7:941–6. 10.1038/90984 11479627

[35] Yamamoto S Matsushita Y Nakagawa T Hayashi T Noda M Mizoue T. Circulating adiponectin levels and risk of type 2 diabetes in the Japanese. Nutr Diabetes. (2014) 4:e130. 10.1038/nutd.2014.27 25133442 PMC4151175

[36] Manzo ND LaGier AJ Slade R Ledbetter AD Richards JH Dye JA. Nitric oxide and superoxide mediate diesel particle effects in cytokine-treated mice and murine lung epithelial cells—implications for susceptibility to traffic- related air pollution. Part Fibre Toxicol. (2012) 9:43. 10.1186/1743-8977-9-43 23151036 PMC3546033

[37] Uski OJ Happo MS Jalava PI Brunner T Kelz J Obernberger I. Acute systemic and lung inflammation in C57Bl/6J mice after intratracheal aspiration of particulate matter from small-scale biomass combustion appliances based on old and modern technologies. Inhal Toxicol. (2012) 24:952–65. 10.3109/08958378.2012.742172 23216156

[38] Manzetti S Andersen O. Biochemical and physiological effects from exhaust emissions. A review of the relevant literature. Pathophysiology. (2016) 23:285–93. 10.1016/j.pathophys.2016.10.002 27793419

[39] Grundy SM Cleeman JI Daniels SR Donato KA Eckel RH Franklin BA. Diagnosis and management of the metabolic syndrome: an american heart association/national heart, lung, and blood institute scientific statement. Circulation. (2005) 112:2735–5. 10.1161/CIRCULATIONAHA.105.169404 16157765

[40] Huang W Wang L Li J Liu M Xu H Liu S. Short-term blood pressure responses to ambient fine particulate matter exposures at the extremes of global air pollution concentrations. Am J Hypertens. (2018) 31:590–99. 10.1093/ajh/hpx216 29409056 PMC5905592

[41] Liu S Brook RD Huang W Fan Z Xu H Wu R. Extreme levels of ambient air pollution adversely impact cardiac and central aortic hemodynamics: the AIRCMD-China study. J Am Soc Hypertens. (2017) 11:754–61.e3. 10.1016/j.jash.2017.09.009 29031802

[42] Dabass A Talbott EO Rager JR Marsh GM Venkat A Holguin F Systemic inflammatory markers associated with cardiovascular disease and acute and chronic exposure to fine particulate matter air pollution (PM2.5) among US NHANES adults with metabolic syndrome. Environ Res. (2018) 161:485–91. 10.1016/j.envres.2017.11.042 29223110

[43] Wei Y Zhang JJ Li Z Gow A Chung KF Hu M. Chronic exposure to air pollution particles increases the risk of obesity and metabolic syndrome: findings from a natural experiment in Beijing. FASEB J. (2016) 30:2115–22. 10.1096/fj.201500142 26891735 PMC6137545

[44] Eze IC Schaffner E Foraster M Imboden M von Eckardstein A Gerbase MW. Long-term exposure to ambient air pollution and metabolic syndrome in adults. PLoS ONE. (2015) 10:e0130337. 10.1371/journal.pone.0130337 26103580 PMC4478007

[45] Hug C Wang J Ahmad NS Bogan JS Tsao TS Lodish HF. T-cadherin is a receptor for hexameric and high-molecular-weight forms of Acrp30/adiponectin. Proc Natl Acad Sci USA. (2004) 101:10308–13. 10.1073/pnas.0403382101 15210937 PMC478568

[46] Imboden M Kumar A Curjuric I Adam M Thun GA Haun M. Modification of the association between PM10 and lung function decline by cadherin 13 polymorphisms in the SAPALDIA cohort: a genome-wide interaction analysis. Environ Health Perspect. (2015) 123:72–9. 10.1289/ehp.1307398 25127211 PMC4286270

[47] Lee S Park H Kim S Lee EK Lee J Hong YS. Fine particulate matter and incidence of metabolic syndrome in non-CVD patients: a nationwide population-based cohort study. Int J Hyg Environ Health. (2019) 222:533–40. 10.1016/j.ijheh.2019.01.010 30797734

[48] Yang BY Qian Z Howard SW Vaughn MG Fan SJ Liu KK. Global association between ambient air pollution and blood pressure: a systematic review and meta-analysis. Environ Pollut. (2018) 235:576–88. 10.1016/j.envpol.2018.01.001 29331891

[49] Molfino A Muscaritoli M Germano A Ramaccini C Alfano R Spagnoli A Changes in metabolic, nutritional and hormonal profile among subjects professionally exposed to urban pollution in a high intensity traffic area. J Cachexia Sarcopenia Muscle. (2019) 10:1378–435. 10.1002/jcsm.12420

